# Re-examination of the taxonomic status of *Enterobacter helveticus*, *Enterobacter pulveris* and *Enterobacter turicensis* as members of the genus *Cronobacter* and their reclassification in the genera *Franconibacter* gen. nov. and *Siccibacter* gen. nov. as *Franconibacter helveticus* comb. nov., *Franconibacter pulveris* comb. nov. and *Siccibacter turicensis* comb. nov., respectively

**DOI:** 10.1099/ijs.0.059832-0

**Published:** 2014-10

**Authors:** Roger Stephan, Christopher J. Grim, Gopal R. Gopinath, Mark K. Mammel, Venugopal Sathyamoorthy, Larisa H. Trach, Hannah R. Chase, Séamus Fanning, Ben D. Tall

**Affiliations:** 1 Institute for Food Safety and Hygiene, University of Zurich, Zurich, Switzerland; 2 CFSAN, FDA, Laurel, USA; 3 UCD Centre for Food Safety, School of Public Health, Physiotherapy & Population Science, University College, Dublin, Ireland; 4 WHO Collaborating Centre for *Cronobacter*, Belfield, Dublin, Ireland

## Abstract

Recently, a taxonomical re-evaluation of the genus *Enterobacter*, based on multi-locus sequence typing (MLST) analysis, has led to the proposal that the species *Enterobacter pulveris*, *Enterobacter helveticus* and *Enterobacter turicensis* should be reclassified as novel species of the genus *Cronobacter*. In the present work, new genome-scale analyses, including average nucleotide identity, genome-scale phylogeny and k-mer analysis, coupled with previously reported DNA–DNA hybridization values and biochemical characterization strongly indicate that these three species of the genus *Enterobacter* are not members of the genus *Cronobacter*, nor do they belong to the re-evaluated genus *Enterobacter*. Furthermore, data from this polyphasic study indicated that all three species constitute two new genera. We propose reclassifying *Enterobacter pulveris* and *Enterobacter helveticus* in the genus *Franconibacter* gen. nov. as *Franconibacter pulveris* comb. nov. (type strain 601/05^T^ = LMG 24057^T^ = DSM 19144^T^) and *Franconibacter helveticus* comb. nov. (type strain 513/05^T^ = LMG 23732^T^ = DSM 18396^T^), respectively, and *Enterobacter turicensis* in the genus *Siccibacter* gen. nov. as *Siccibacter turicensis* comb. nov. (type strain 508/05^T^ = LMG 23730^T^ = DSM 18397^T^).

Stephan *et al.* (2007, 2008) reported three novel species, *Enterobacter pulveris*, *Enterobacter helveticus* and *Enterobacter turicensis* isolated from dried fruit powders, powdered infant formula (PIF), a number of PIF-production environments and other dried food ingredients. These species of the genus *Enterobacter* were originally isolated during initial work being carried out to define the genus *Cronobacter*, as described by Iversen *et al.* (2007, 2008). The original decision used to justify their exclusion from the genus *Cronobacter* was clearly based on differences in their phenotypic characteristics, as well as data from DNA–DNA hybridization and the phylogenetic analysis of the *rpoB* gene (Stephan *et al.*, 2007, 2008). However, these novel species do share several phenotypic and metabolic characteristics with members of the genus *Cronobacter*, such as resistance to desiccation, production of a yellow Pantoea-like, carotenoid pigment (Lehner *et al.*, 2006) and constitutive metabolism of 5-bromo-4-chloro-3-indolyl-α-d-glucopyranoside, which is the feature used in the differentiation of presumptive colonies of members of the genus *Cronobacter* growing on most chromogenic Cronobacter isolation agars (Iversen *et al.*, 2004).

There is no indication that *Enterobacter pulveris*, *Enterobacter helveticus* and *Enterobacter turicensis* pose a threat to public health. In contrast, it is well-documented that members of the genus *Cronobacter* (except for the single species *Cronobacter condimenti*) are opportunistic foodborne pathogens and known to be rare, but important, causes of invasive life-threatening neonatal and infantile infections; which can lead to severe disease manifestations such as brain abscesses, meningitis, necrotizing enterocolitis and systemic sepsis (Bowen & Braden, 2006).

Recently, Brady *et al.* (2013) re-evaluated the taxonomy of the genus *Enterobacter*, based primarily on multi-locus sequence analysis (MLSA) by partial sequencing of four housekeeping genes (*gyrB, rpoB, infB* and *atpD*), and these authors proposed that *Enterobacter helveticus*, *Enterobacter pulveris* and *Enterobacter turicensis* should be recognized as species of the genus *Cronobacter*. The authors asserted that phylogenetic analysis of the concatenated nucleotide sequences of these four genes provided differentiation between previously described members of the genus *Enterobacter*; grouping them into five strongly supported MLSA groups. MLSA group E included the seven described species of the genus *Cronobacter* along with *Enterobacter turicensis*, *Enterobacter helveticus* and *Enterobacter pulveris*. A closer examination of the phylogenetic tree from this study, however, reveals that MLSA group E consists of two well-differentiated clades; one of which contains the seven well recognized species of the genus *Cronobacter* and the second clade consisting of two subclades, one containing *Enterobacter helveticus* and *Enterobacter pulveris* and the other containing *Enterobacter turicensis*.

In an effort to further clarify the taxonomic standing of these three species, we performed genome-scale analyses using whole-genome sequencing data from multiple strains from each species, to augment previously reported genotypic and phenotypic results (Stephan *et al.*, 2007, 2008). By reapplying this polyphasic approach to include new whole-genome sequence data, these data clarify the taxonomic standing of these species. Average nucleotide identity (ANI) (Goris *et al.*, 2007), by blast, was computed using the JSpecies package (Richter and Roselló-Móra, 2009). 16S rRNA gene sequence phylogeny, using partial sequences downloaded from the NCBI GenBank repository and representative of the seven type strains of species of the genus *Cronobacter*, *Enterobacter cloacae* and the six newly assembled genomes of *Enterobacter pulveris*, *Enterobacter turicensis* and *Enterobacter helveticus* was computed after alignment with clustal w using the mega5 phylogeny suite (Tamura *et al.*, 2011). The tree was generated using the maximum-likelihood method.

Genome-scale phylogeny was computed using a single-nucleotide polymorphism (SNP)-based approach: 23 genomes of members of the genus *Cronobacter*, 12 whole genome assemblies for members of the genera *Klebsiella*, *Escherichia*, *Citrobacter* and *Salmonella*, available at NCBI, and assembled genomes of *Enterobacter turicensis*, *Enterobacter helveticus* and *Enterobacter pulveris* reported by Grim *et al.* (2013), Gopinath *et al.* (2013) and Stephan *et al.* (2013) were used to create a local blast database. Using *Cronobacter sakazakii* BAA-894 as the reference strain, this database was queried using in-house Perl scripts (Perl scripts will be made available upon request). A SNP-profile was generated for 300 randomly chosen BAA-894 homologues found among the 51 genomes and used to create a phylogram using mega software version 5 (Tamura *et al.*, 2011). This phylogram was then used to compare the genomes of species of the genus *Cronobacter* with the six *Enterobacter pulveris*, *Enterobacter helveticus* and *Enterobacter turicensis* strains using a novel k-mer analysis scheme. A k-mer is a motif of a coding sequence in a genomic sequence and it is defined by its oligonucleotide size and frequency distribution within a genome. K-mers are not necessarily found more than once in a genome and this is characteristic of analyses developed for k-mers of 25 oligonucleotides or more. The analysis was carried out by developing a database of 25-mers for each sequenced strain, then by computationally identifying unique and shared k-mers among the strains of the two genera.

PCR analysis for the prevalence of plasmid-encoded virulence factor genes (plasmidotyping) and other genotyping assays such as PCR analysis for the presence of the *Cronobacter*-specific *zpx* (zinc metalloprotease) gene and species-specific *cgcA* (diguanylate cyclase) and *rpoB* genes were performed as described previously by Franco *et al.* (2011), Kothary *et al.* (2007), Carter *et al.* (2013), Stoop *et al.* (20 nd Lehner *et al.* (2012).

16S rRNA gene sequence phylogeny is shown in Fig. 1 and is in agreement with previous partial and full-length 16S rRNA gene sequencing results for members of the genus *Cronobacter* as described by Iversen *et al.* (2007) and *Enterobacter helveticus*, *Enterobacter pulveris* and *Enterobacter turicensis* (Stephan *et al.* (2007, 2008). The 16S rRNA gene sequence-based tree of the tested members of the family *Enterobacteriaceae* appears not to be reflective of the phylogenetic relationships among the species and genera as observed using other methods. In addition, the resolution of the tree is not sufficient to capture the subtle differences among species of the genus *Cronobacter* and these related species.

**Fig. 1.**
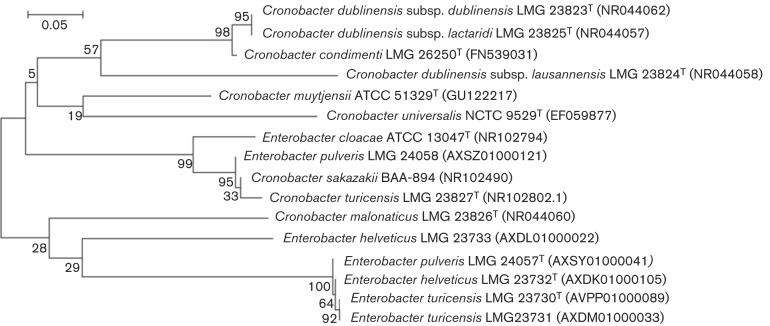
Phylogenetic tree of partial 16S rRNA gene sequences of type species from the genus *Cronobacter*, *Enterobacter cloacae*, *Enterobacter turicensis*, *Enterobacter helveticus* and *Enterobacter pulveris*. The rRNA gene sequences were downloaded from the NCBI GenBank repository and were aligned with clustal w using the mega5 phylogeny suite (Tamura *et al.* 2011). The tree was generated using maximum-likelihood algorithm. The bootstrap values obtained from 1000 bootstrap replicates are reported as percentages at the nodes. Bar, 0.05 changes per nucleotide value.

DNA–DNA hybridization results are summarized in Table S1 (available in the online Supplementary Material) and as reported originally by Stephan *et al.* (2007, 2008) clearly indicate that all three species of the genus *Enterobacter* investigated in this study are in fact three distinct species, as comparisons among the strains of each proposed species fall well below the accepted 70 % DNA–DNA relatedness threshold. Furthermore, the results indicate that the two strains of *Enterobacter helveticus*, 513/05^T^ and 1159/04 (100 % DNA–DNA relatedness), and the two strains of *Enterobacter pulveris*, 1160/04 and 601/05^T^ (99–100 %), are highly clonal. The two strains of *Enterobacter turicensis*, 508/05^T^ and 610/05, were also highly related to one another (95 %). These results are probably explained by the fact that each pair of strains was isolated from a similar desiccated, powdered food or food production environment.

Unfortunately, DNA–DNA hybridization studies are not generally utilized to delineate genera boundaries. From Table S1, it is clear that the two strains of *Enterobacter pulveris* and *Enterobacter helveticus* are more closely related to each other (54 %) than either is to  *Enterobacter turicensis* (23–27 %). From our previous work, we found that the DNA–DNA hybridization values are consistent with those among species of the same genus (Iversen *et al.*, 2008), in this case, the genus *Cronobacter*.

ANI has emerged as one of the predominant genomic alternatives to DNA–DNA hybridization. We conducted numerous pairwise ANI analyses between genomes of *Enterobacter pulveris*, *Enterobacter turicensis*, *Enterobacter helveticus* and members of the genera *Cronobacter* and *Enterobacter*, as well as other enteric bacteria (Table S2). The pairwise ANI values between each of the two strains of the three species of the genus *Enterobacter* are in excellent agreement with the DNA–DNA hybridization values. An ANI value of 95 % has been set as a species threshold, corresponding to a DNA–DNA hybridization value of 70 % (Goris *et al.*, 2007). The current species epithet designations among the six isolates of members of the genus *Enterobacter* examined in this study are in agreement with this threshold (Table S2). This analytical approach would support efforts aimed at clarifying taxonomic relationships within the core members of the genus *Enterobacter* as well (Table S2).

While ANI has been extensively applied to the examination of species delineation, we propose that this analysis can be extremely informative in questions regarding genus-level demarcations. We included several species of two genera, *Cronobacter* and the core *Enterobacter* group, in our ANI analyses. In both cases, the minimum pairwise ANI values between species within each genus are greater than 85 %. Other pairwise species ANI values in Table S2, such as between *Dickeya dianthicola* strain IPO 980 and *Dickeya solani* strain MK10 (91.8 %), or *Citrobacter freundii* strain 4_7_47CFAA and *Citrobacter* spp. strain KTE151 (92.1 %), fall within this threshold. Still other pairwise comparisons; for example, *Klebsiella pneumoniae* KPNIH1 and *Klebsiella mobilis* (*Enterobacter aerogenes*) FG135 (84.5 %), or *Enterobacter cloacae* subsp. *dissolvens* SP1 and *Kosakonia radicincitans* DSM 16656 (83.5%), indicate that this threshold should in fact be lower, or that these taxonomic relationships need to be re-examined more closely. Interestingly, strains of *Enterobacter helveticus* and *Enterobacter pulveris* have pairwise ANI values that support the inclusion of these two species in one genus, while pairwise ANI values between *Enterobacter turicensis* and this group indicate that they are indeed two distinct genus-level taxonomic groups (Table S2).

Although pairwise ANI values provide a benchmark of divergence (or similarity) between two genomes, evolutionary relationships between more than two genomes cannot be inferred from this analysis. Therefore, genome-scale phylogenetic analysis using SNP profiles (Fig. 2) and k-mer analysis was performed. When 300 random genes of *Cronobacter sakazakii* strain BAA-894 were used to assess for the presence of SNPs in their homologues from each of the 40 other enteric genomes, a stable phylogenetic profile emerged. This approach allowed for the repeated examination of any random number of genes for validation, and in each case a top-level, five-clade phylogram pattern emerged. Fig. 2 shows a representative tree in which five major clades were noted. In clade I, strains from all seven species of the genus *Cronobacter* with validly published names (Iversen *et al.*, 2008; Joseph *et al.*, 2012) grouped together. The two genomes of strains of *Enterobacter cloacae* used in this analysis grouped within clade III, along with isolates of the genera *Citrobacter*, *Salmonella*, *Escherichia* and *Klebsiella*. Lastly, the isolates of members of the genus *Pantoea* grouped within clade V. Interestingly, the two strains of *Enterobacter turicensis* grouped separately in clade IV, being distinct from the clade containing the *Enterobacter helveticus* and *Enterobacter pulveris* strains, which grouped into clade II. These results unambiguously confirm that these six isolates classified as members of the genus *Enterobacter* are not members of the genus *Cronobacter* nor of the genus *Enterobacter*, and furthermore, these data indicate that these bacteria should be placed into two new unique genus-level taxonomic groups.

**Fig. 2.**
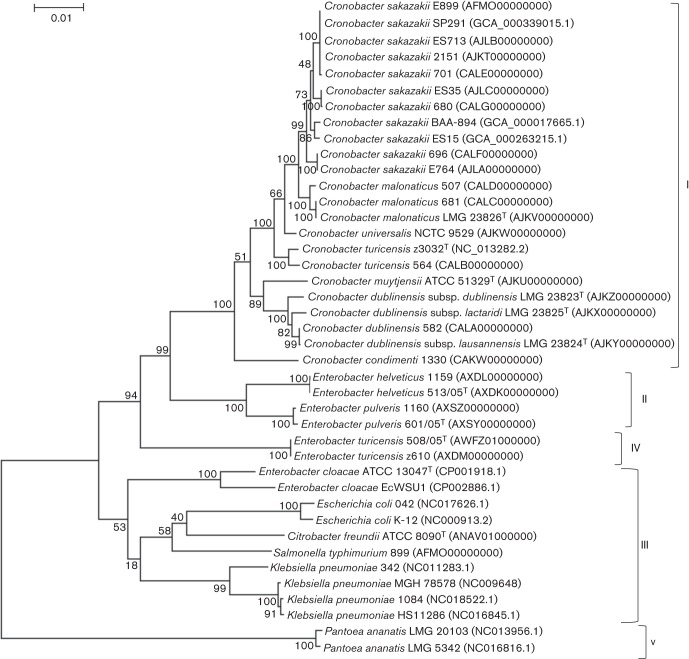
Evolutionary relationships of 23 members of the genus *Cronobacter*, 10 members of the genus *Enterobacter* and eight genomes of members of related genera. Clades are represented by roman numerals I–V. Clade I represents genomes from 23 strains of members of the genus *Cronobacter*, Clade II represents genomes from strains of *Enterobacter helveticus* and *Enterobacter pulveris*. Clade III represents genomes from three strains of *Enterobacter cloacae*, genomes from members of the genera *Citrobacter*, *Salmonella*, *Escherichia* and *Klebsiella*. Clade IV represents genomes from strains of *Enterobacter turicensis*. Lastly, genomes from two strains of a member of the genus *Pantoea* are represented in clade V. Neighbour-joining phylogeny is based on the alignment of SNPs from 300 orthologous genes. The bootstrap values obtained from 1000 bootstrap replicates are reported as percentages at the nodes. The tree is drawn to scale, with branch lengths in the same units as those of the evolutionary distances used to infer the phylogenetic tree. Bar, 0.01 base substitutions per site.

Further genomic analyses using k-mer signatures confirmed and supported these observations. Based on the above phylogram, k-mer signature sets were generated for each group of genomes. Over 29 000 unique k-mer signatures for *Cronobacter sakazakii*, approximately 67 000 signatures for *Cronobacter malonaticus*, 36 500 signatures for *Cronobacter turicensis*, 64 000 for *Cronobacter muytjensii* and 36 500 signatures for *Cronobacter dublinensis* were generated at genus-level for the genus *Cronobacter* (clade I). In contrast, k-mer analysis for *Enterobacter helveticus* and *Enterobacter pulveris* together (clade II) contained over 166 000 signatures compared with approximately 152 500 signatures for *Enterobacter turicensis* (clade IV). When all the genomes from clade I were combined for the genus *Cronobacter* clade (genus-specific k-mers), the number of k-mer signatures was approximately 6000 compared with approximately 14 000 k-mer signatures for the strains of species of the genus *Enterobacter* in clades II and IV. Subsequently, k-mer analysis showed that there were only 908 k-mer signatures in common between all genomes of members of the genus *Cronobacter* in clade I and the genomes of members of the genus *Enterobacter* in clades II and IV. Together, these results indicate that *Enterobacter helveticus*, *Enterobacter pulveris* and *Enterobacter turicensis* are genomically distinct from any member of the genus *Cronobacter*; that the members of the *Enterobacter helveticus*/*Enterobacter pulveris* clade are more related to each other than to either of the *Enterobacter turicensis* isolates; and these three species of the genus *Enterobacter* may represent two distinct taxonomic groups, as shown in the phylogram in Fig. 2.

Phenotypic characteristics that differentiate *Enterobacter turicensis*, *Enterobacter pulveris* and *Enterobacter helveticus* from species of the genus *Cronobacter* were based on results from ID 32 E and API 20 E tests (bioMérieux) and Biolog phenotypic microarray analyses (Biolog) as reported by Stephan *et al.* (2007, 2008), Iversen *et al.* (2008) and Joseph *et al.* (2012). Based on their corresponding phenotypes, this phylogenetic placement is further supported by the fact that *Enterobacter pulveris* and *Enterobacter helveticus* can utilize 5-keto-d-gluconate, trans-aconitate, quinate, *p*-hydroxybenzoate and protocatehuate while *Enterobacter turicensis* cannot utilize these substrates. Also by using the API 20 E test kit and the Vitek 2.0 Compact GN card (Vitek software version 5), *Enterobacter turicensis*, *Enterobacter pulveris* and *Enterobacter helveticus* are ornithine decarboxylase (ODC)-negative (Table 1). Biochemically, *Enterobacter turicensis*, *Enterobacter helveticus* and *Enterobacter pulveris* can be clearly differentiated from members of the genus *Cronobacter*, as shown in Table 1, by the following common phenotypic characteristics: ODC, Voges–Proskauer test, methyl red test, arginine dihydrolase, mucate utilization and palatinose utilization (Iversen *et al.*, 2008; Stephan *et al.*, 2007, 2008). Key biochemical reactions which differentiate *Enterobacter pulveris* from *Enterobacter helveticus* are the ability of *Enterobacter pulveris* to ultiize sucrose, d-arabitol, raffinose and fumerate as a carbon source and to produce acid from cellobiose, d-arabitol, sucrose and l-rhamnose, whereas *Enterobacter helveticus* cannot utilize or produce acid from these substrates. Furthermore, lytic phages targeting the different species of the genus *Cronobacter* do not lyse *Enterobacter turicensis*, *Enterobacter helveticus* or *Enterobacter pulveris* (not shown).

**Table 1.** Phenotypic characteristics that differentiate *Siccibacter* *turicensis* comb. nov., *Franconibacter* *pulveris* comb. nov. and *Franconibacter* *helveticus* comb. nov. from species of the genus *Cronobacter* Taxa: 1, *Siccibacter* *turicensis* comb. nov.; 2, *Franconibacter* *pulveris* comb. nov.; 3, *Franconibacter* *helveticus* comb. nov.; 4, *Cronobacter condimenti*; 5, *Cronobacter universalis*; 6, *Cronobacter sakazakii*; 7, *Cronobacter malonaticus*; 8, *Cronobacter turicensis*; 9, *Cronobacter muytjensii*; 10, *Cronobacter dublinensis* subsp. *dublinensis*; 11, *Cronobacter dublinensis* subsp. *lactaridi*; 12, *Cronobacter dublinensis* subsp. *lausannensis*. Data for taxa 4–12 taken from Stephan *et al.* (2007, 2008), Iversen *et al.* (2008) and Joseph *et al.* (2012). All strains were negative for D-sorbitol, L-fucose and 3-*O*-methyl-D-glucopyranose. +, Positive; −, negative; v, variable; nd, no data available.

Characteristic	1	2	3	4	5	6	7	8	9	10	11	12
Voges–Proskauer	−	−	−	+	+	+	+	+	+	+	+	+
Methyl red test	+	+	+	−	−	−	−	−	−	−	−	−
Ornithine decarboxylase	−	−	−	+	+	+	+	+	+	+	+	+
Arginine dihydrolase	−	−	−	+	+	+	+	+	+	+	+	+
Motility	+	+	+	−	−	+	+	+	+	+	+	+
Carbon source utilization												
Malonate	+	−	+	+	+	−	+	+	+	+	−	−
Sucrose	−	+	−	+	+	+	+	+	+	+	+	+
d-Arabitol	−	+	−	−	−	−	−	−	−	−	−	−
Mucate	+	+	+	−	−	−	−	−	−	−	−	−
Dulcitol	+	v	+	−	+	−	−	+	+	−	−	−
Putrescine	−	v	+	−	−	+	+	+	+	+	+	v
α-l-Rhamnose	+	+	+	+	+	+	+	+	+	+	+	+
Raffinose	−	+	−	+	+	+	+	+	+	+	+	+
*trans*-aconitate	−	+	+	−	−	−	+	−	v	+	+	+
5-Keto-d-Gluconate	−	+	+	−	−	−	−	−	−	−	−	−
Fumarate	−	+	−	nd	+	+	+	+	+	+	+	+
Quinate	−	+	+	nd	−	−	−	−	−	−	−	−
*p*-Hydroxybenzoate	−	+	+	nd	−	−	−	−	−	−	−	−
Protocatechuate	−	+	+	nd	−	−	−	−	−	−	−	−
Turanose	+	v	+	−	−	+	+	+	v	+	v	−
Acid production from:												
Palatinose	−	−	−	+	+	+	+	+	+	+	+	+
Cellobiose	−	+	−	+	+	+	+	+	+	+	+	+
d-Arabitol	−	+	−	−	−	−	−	−	−	−	−	−
Sucrose	−	+	−	+	+	+	+	+	+	+	+	+
l-Rhamnose	+	+	−	+	+	+	+	+	+	+	+	+

Genotypic analysis using PCR-based assays for the detection of targets specific to members of the genus *Cronobacter* included α-glucosidase, *rpoB*, *zpx* (zinc metalloprotease) and *cgcA* (diguanylate cyclase) genes as described by Iversen *et al.* (2007), Stoop *et al.* (2009), Lehner *et al.* (2012), Kothary *et al.* (2007) and Carter *et al.* (2013). Results of these analyses among strains of *Enterobacter turicensis*, *Enterobacter helveticus* and *Enterobacter pulveris* showed that these strains were negative for these gene targets, further extending support for our phylogenetic findings. In addition plasmidotyping studies, as described by Franco *et al.* (2011), showed that only *Enterobacter turicensis* strains 610/05 and 508/05^T^ contained a plasmid of the IncF1B type with a *repA* replication gene, and all other plasmid gene targets for members of the genus *Cronobacter* were not identified by PCR analysis (Grim *et al.*, 2013). This analysis also demonstrated that this plasmid lacked the two iron-acquisition systems, which together comprise gene clusters of the common virulence plasmids of members of the genus *Cronobacter*.

Comparative genomics also revealed a number of distinguishing genotypic characteristics, including several species- and group-specific chaperone/usher fimbriae, bacteriophage, or prophage-like elements, plasmids, transposons and several metabolic traits (Grim *et al.*, 2013; Gopinath *et al.*, 2013; Stephan *et al.*, 2013). Specifically, both strains of *Enterobacter turicensis* used in this study possessed a type III secretion system and two conjugative plasmids. Conversely, both strains of *Enterobacter pulveris* and *Enterobacter helveticus* possessed operons for the catabolism of l-idonate, an unspecified β-xyloside, putrescine, fructose and lysine, as well as the *pga* biofilm operon and the *lsr* autoinducer-2 operon. Between these two species, and of note, the two strains of *Enterobacter pulveris* possessed operons for the catabolism of sialic acid (*nan*), mannitol/arabitol and sucrose, and identical CRISPR elements, while the two strains of *Enterobacter helveticus* possessed a unique homologous maltose 6-phosphate utilization operon, as well as a haemin ABC transporter. Interestingly, *Enterobacter helveticus* strain 1159/04 harboured a smaller plasmid homologous to IncN2 plasmids shown to carry the New Delhi metallo-β-lactamase (NDM-1)-encoding gene (Chen *et al.*, 2012; Grim *et al.*, 2013; Poirel, *et al.*, 2011).

Kothary *et al.* (2007) have reported that members of the genus *Cronobacter* contained a zinc metalloprotease gene, *zpx* and that the nucleotide region encompassing the conserved zinc-binding site was a useful genus-specific target for the detection of members of the genus *Cronobacter*. Kothary *et al.* (2007) showed that the advantage of this genus-specific assay is that these closely related species of the genus *Enterobacter* were differentiated from members of the genus *Cronobacter* because they do not yield the 350 bp amplicon. Phylogenetic analysis of *zpx* sequences, shown in Fig. S1 demonstrates that these three species of the genus *Enterobacter* possessed related, but distinct *zpx* orthologues, which strengthens the support for the distinct and separate taxonomic relatedness of these species proposed on the basis of the genome-scale phylogenetic analyses described in Fig. 2.

Therefore, since no minimal requirements for genus characterization exist (Wayne *et al.*, 1987) and based on the genomic and phenotypic data reported here, we propose reclassifying *Enterobacter turicensis* in a new genus named *Siccibacter* gen. nov., which is to separate this species from the genera *Cronobacter* and *Enterobacter*. It is also proposed that *Enterobacter helveticus* and *Enterobacter pulveris* are reclassified in a separate genus, named *Franconibacter* gen. nov.

## Description of *Siccibacter* gen. nov.

*Siccibacter* (Sic.ci.bac′ter. L. adj. *siccus* dry; N.L. masc. n. *bacter* rod; N.L. masc. n. *Siccibacter* dry rod).

The description is based on that of Stephan *et al.* (2007). Cells are 1.0 µm wide and 1.5–2.5 µm long Gram-reaction-negative peritrichously flagellated, coccoid to rod-shaped and occur singly or in pairs. They are weakly oxidase-positive, catalase-positive and facultatively anaerobic. After 24 h of aerobic incubation at 37 °C on TSA medium, colonies are yellow-pigmented and convex. Colonies grow well at 10 °C (within 3 days) but poorly at 44 °C. Positive for malonate and negative for urease, arginine dihydrolase and ornithine and lysine decarboxylase. Tests for indole and H_2_S production and the Voges–Proskauer reaction are negative. Acid is produced from the following compounds: galacturonate, d-mannitol, maltose, d-glucose, l-arabinose, trehalose and l-rhamnose. No acid production is observed from l-arabitol, d-arabitol, 5-ketogluconate, sodium pyruvate, adonitol, palatinose, sucrose, inositol, cellobiose or d-sorbitol. The chromogenic substrates ONPG, 4-nitrophenyl β-d-glucopyranoside, 4-nitrophenyl β-d-galactopyranoside, 4-nitrophenyl α-d-glucopyranoside, 4-nitrophenyl α-d-galactopyranoside and 4-nitrophenyl α-d-maltopyranoside are hydrolysed. The following compounds are not hydrolysed: 5-bromo-3-indoxyl-nonanoate, 4-nitrophenyl β-d-glucuronide, palatinose and l-aspartic acid 4-nitroanilide. Positive reaction in tests for the utilization of α-d-glucose, β-d-fructose, d-galactose, trehalose, d-mannose, α-melibiose, maltotriose, maltose, α-lactose, 1-*O*-methyl β-galactopyranoside, 1-*O*-methyl α-galactopyranoside, cellobiose, β-gentiobiose, 1-*O*-methyl β-d-glucopyranoside, aesculin, d-ribose, l-arabinose, d-xylose, α-l-rhamnose, dulcitol, glycerol, d-mannitol, turanose, d-saccharate, mucate, l-malate, *cis*-aconitate, d-glucuronate, d-galacturonate, 2-keto-d-gluconate, *N*-acetyl-d-glucosamine, d-gluconate, dl-lactate, d-glucosamine, l-aspartate, l-glutamate, l-proline, l-alanine and l-serine. The following compounds are not utilized as sole sources of carbon: l-sorbose, sucrose, raffinose, lactulose, α-l-fucose, d-arabitol, l-arabitol, xylitol, d-tagatose, *myo*-inositol, maltitol, d-sorbitol, adonitol, hydroxyquinoline-β-glucuronide, i-erythritol, 1-*O*-methyl α-d-glucopyranoside, 3-*O*-methyl d-glucopyranose, l-tartrate, d-tartrate, *myo*-tartrate, *trans*-aconitate, tricarballylate, 5-keto-d-gluconate, l-tryptophan, phenylacetate, protocatechuate, *p*-hydroxybenzoate, quinate, gentisate, *m*-hydroxybenzoate, benzoate, 3-phenylpropionate, trigonelline, betain, putrescine, dl-amino-*N*-butyrate, histamine, caprate, caprylate, l-histidine, fumarate, glutarate, dl-glycerate, dl-α-amino-*N*-valerate, ethanolamine, tryptamine, itaconate, dl-β-hydroxybutyrate, malonate, propionate, l-tyrosine or 2-oxoglutarate. 

The type species is *Siccibacter turicensis*.

## Description of *Siccibacter turicensis* comb. nov.

*Siccibacter turicensis* (tu.ric.en′sis. L. masc. adj. *turicensis* from Turicum/Zurich, from where the species was first isolated).

Basonym: *Cronobacter zurichensis* (Stephan *et al.* 2007) Brady *et al.* 2013.

The description of this taxon is the same as that given by Stephan *et al.* (2007) for *Enterobacter turicensis*.

The type strain is 508/05^T^ ( = LMG 23730^T^ = DSM 18397^T^). The draft genome assembly of the type strain LMG 23730^T^ has a size of 4 183 714 bp and a DNA G+C content of 58.0 % (Stephan *et al.*, 2013).

## Description of *Franconibacter* gen. nov.

*Franconibacter* (Fran.co.ni.bac′ter. N.L. masc. n. *bacter* a rod; N.L. masc. n. *Franconibacter* a rod named in memory of microbiologist Augusto Franco-Mora).

The description is based on those of Stephan *et al.* (2007, 2008).

Gram-reaction-negative coccoid to rod-shaped that are facultatively anaerobic and motile. Cells are 0.9–1.0 µm wide by 1.5–3.0 µm long and occur singly or in pairs. After 24 h aerobic incubation at 37 °C on TSA medium, colonies are yellow-pigmented and convex. Catalase-positive and negative or weakly positive for oxidase. After 24 h of aerobic incubation at 37 °C on TSA medium, colonies are yellow pigmented and convex. Colonies grow poorly at 10 °C (within 3 days), but grow well at 44 °C. Positive for the hydrolysis of 5-bromo-3-indoxyl-nonanoate and the utilization of trans-aconitate, 5-keto-d-gluconate, protocatechuate, *p*-hydroxybenzoate and quinate. Negative result in tests for urease and ornithine decarboxylase, arginine dihydrolase and lysine decarboxylase activities, indole and H_2_S production and the Voges–Proskauer reaction. Acid is produced from the following compounds: galacturonate, d-mannitol, maltose, d-glucose, l-arabinose and trehalose. No acid production is observed for l-arabitol, 5-ketogluconate, sodium pyruvate, adonitol, palatinose, inositol or d-sorbitol. The chromogenic substrates ONPG, 5-bromo-3-indoxyl-nonanoate, 4-nitrophenyl β-d-glucopyranoside, 4-nitrophenyl β-d-galactopyranoside, 4-nitrophenyl α-d-glucopyranoside, 4-nitrophenyl α-d-galactopyranoside and 4-nitrophenyl α-d-maltopyranoside are hydrolysed. The following compounds are not hydrolysed: 4-nitrophenyl β-d-glucuronide and l-aspartic acid 4-nitroanilide. Positive reaction in tests for the utilization of α-d-glucose, β-d-fructose, d-galactose, trehalose, d-mannose, α-melibiose, maltotriose, maltose, α-lactose, 1-*O*-methyl β-galactopyranoside, 1-*O*-methyl α-galactopyranoside, cellobiose, β-gentiobiose, 1-*O*-methyl β-d-glucopyranoside, d-ribose, l-arabinose, d-xylose, α-l-rhamnose, glycerol, d-mannitol, d-saccharate, mucate, l-malate, *cis*-aconitate, *trans*-aconitate, d-glucuronate, d-galacturonate, 2-keto-d-gluconate, 5-keto-d-gluconate, *N*-acetyl-d-glucosamine, d-gluconate, protocatechuate, *p*-hydroxybenzoate, quinate, putrescine, dl-α-amino-*N*-butyrate, dl-lactate, d-glucosamine, l-aspartate, l-glutamate, l-proline, l-alanine and l-serine. The following compounds are not utilized as sole sources of carbon: l-sorbose, α-l-fucose, l-arabitol, xylitol, d-tagatose, *myo*-inositol, maltitol, d-sorbitol, adonitol, hydroxyquinoline-β glucuronide, i-erythritol, 3-O-methyl d-glucopyranose, d-tartrate, myo-tartrate, tricarballylate, l-tryptophan, phenylacetate, gentisate, *m*-hydroxybenzoate, benzoate, 3-phenylpropionate, trigonelline, betain, histamine, caprate, caprylate, l-histidine, glutarate, dl-α-amino-*N*-valerate, ethanolamine, tryptamine, itaconate, dl-β hydroxybutyrate, malonate, propionate, l-tyrosine or 2-oxoglutarate.

The type species is *Franconibacter helveticus*.

## Description of *Franconibacter helveticus* comb. nov.

*Franconibacter helveticus* [hel.ve′ti.cus. L. masc. adj. *helveticus* of Helvetica (Switzerland), from where the species was first isolated].

Basonym: *Cronobacter helveticus* (Stephan *et al.* 2007) Brady *et al.* 2013.

The description of this taxon is the same as that given by Stephan *et al.* (2007) for *Enterobacter helveticus*.

The type strain is 513/05^T^ ( = LMG 23732^T^ = DSM 18396^T^). The draft genome assembly of the type strain *F. helveticus* LMG 23732^T^ ( = 513/05^T^ = DSM 18396^T^) has a size of 4 842 422 bp and a DNA G+C content of 55.4 % (Grim *et al.*, 2013).

## Description of *Franconibacter pulveris* comb. nov.

*Franconibacter pulveris* (pul′ve.ris. L. gen. n. *pulveris* of powder).

Basonym: *Cronobacter pulveris* (Stephan *et al.* 2008) Brady *et al.* 2013.

The description of this taxon is the same as that given by Stephan *et al.* (2008) for *Enterobacter pulveris*.

The type strain is 601/05^T^ ( = LMG 24057^T^ = DSM 19144^T^). The draft genome assembly of the type strain *F. pulveris* LMG 24057^T^ ( = 601/05^T^ = DSM 19144^T^) has a size of 4 708 624 bp and a DNA G+C content of 56.6 % (Gopinath *et al.*, 2013).
